# A Dual‐Phase Approach to Facial Rejuvenation Integrating Contouring and Ligamentous (CL Code) Anchoring With a Polycaprolactone‐Based Collagen Stimulator

**DOI:** 10.1111/jocd.70796

**Published:** 2026-03-31

**Authors:** Gi‐Woong Hong, Isaac Kai Jie Wong, Jovian Wan, Soo Yeon Park, Suyeon Lee, Kyu‐Ho Yi

**Affiliations:** ^1^ Samskin Plastic Surgery Clinic Seoul Korea; ^2^ The Artisan Clinic Private Limited The Paragon Singapore; ^3^ Medical Research Inc. Wonju Korea; ^4^ Made‐Young Plastic Surgery Clinic Korea; ^5^ Division in Anatomy and Developmental Biology, Department of Oral Biology Human Identification Research Institute, BK21 FOUR Project, Yonsei University College of Dentistry Seoul Korea; ^6^ You and I Clinic Seoul Republic of Korea

**Keywords:** collagen/metabolism, face/anatomy and histology, injections, polycaprolactone, rejuvenation/methods, subcutaneous/methods

## Abstract

**Background:**

Facial aging reflects concurrent skeletal remodeling, fat redistribution, and attenuation of retaining ligaments. Volume‐oriented fillers may restore contours but often underaddress structural support.

**Objective:**

To describe the CL Code, a dual‐phase injectable protocol combining Contouring (C) and Ligamentous anchoring (L), using a polycaprolactone (PCL) collagen stimulator to integrate immediate volumization with delayed neocollagenesis.

**Methods:**

The protocol aligned the biphasic kinetics of PCL with facial anatomy. The C‐phase targeted seven contouring zones using small aliquots to restore volume, while the L‐phase reinforced eight retaining ligament support points at the supraperiosteal or subsuperficial musculoaponeurotic plane using 27‐gauge needles. A 49‐year‐old woman with pan‐facial volume loss underwent treatment with a total volume of 2.9 mL (0.3 mL per C site and 0.1 mL per L point). Outcomes at 6 months included Global Aesthetic Improvement Scale (GAIS) ratings and patient‐reported FACE‐Q scores. FACE‐Q was administered pretreatment and at 6 months using the Satisfaction with Facial Appearance module. GAIS was assessed by two independent dermatologists blinded to treatment details using standardized photographs.

**Results:**

In this single patient, standardized photography demonstrated improved jawline definition, reduced jowling, and softening of nasolabial folds. GAIS was rated as “Very Much Improved,” and FACE‐Q scores increased from 58 to 86 (out of 100), indicating high patient satisfaction.

**Conclusions:**

Aligning the temporal behavior of PCL with anatomically targeted injection planes may couple immediate contour restoration with progressive ligamentous support. The CL Code achieved meaningful esthetic improvement using conservative volumes. Controlled comparative studies with longer follow‐up are warranted.

## 1 Introduction

Contemporary understanding of facial aging recognizes a complex interplay of skeletal remodeling, fat compartment redistribution, and attenuation of retaining ligaments [1, 2]. While the distinct mechanisms of immediate volumizers, such as hyaluronic acid fillers, and collagen‐stimulating agents, including polycaprolactone (PCL)‐based products, are well characterized, optimal injection strategies for PCL‐based fillers continue to evolve [3]. Recent clinical studies have demonstrated their efficacy in volumetric restoration; however, the potential to strategically coordinate their biphasic action—comprising an initial carrier gel effect followed by sustained collagen stimulation—with specific anatomical treatment goals warrants further exploration [4, 5, 6].

PCL‐based stimulators offer unique therapeutic advantages through their dual‐phase mechanism. An initial carboxymethylcellulose (CMC) carrier provides immediate volumization lasting approximately 3–6 months, followed by gradual hydrolysis of PCL microspheres that induces progressive neocollagenesis over 12–24 months [4, 7]. Current techniques, while effective for general volumization, may not fully leverage this temporal dissociation to address the hierarchical anatomical changes associated with facial aging.

**FIGURE 1:**
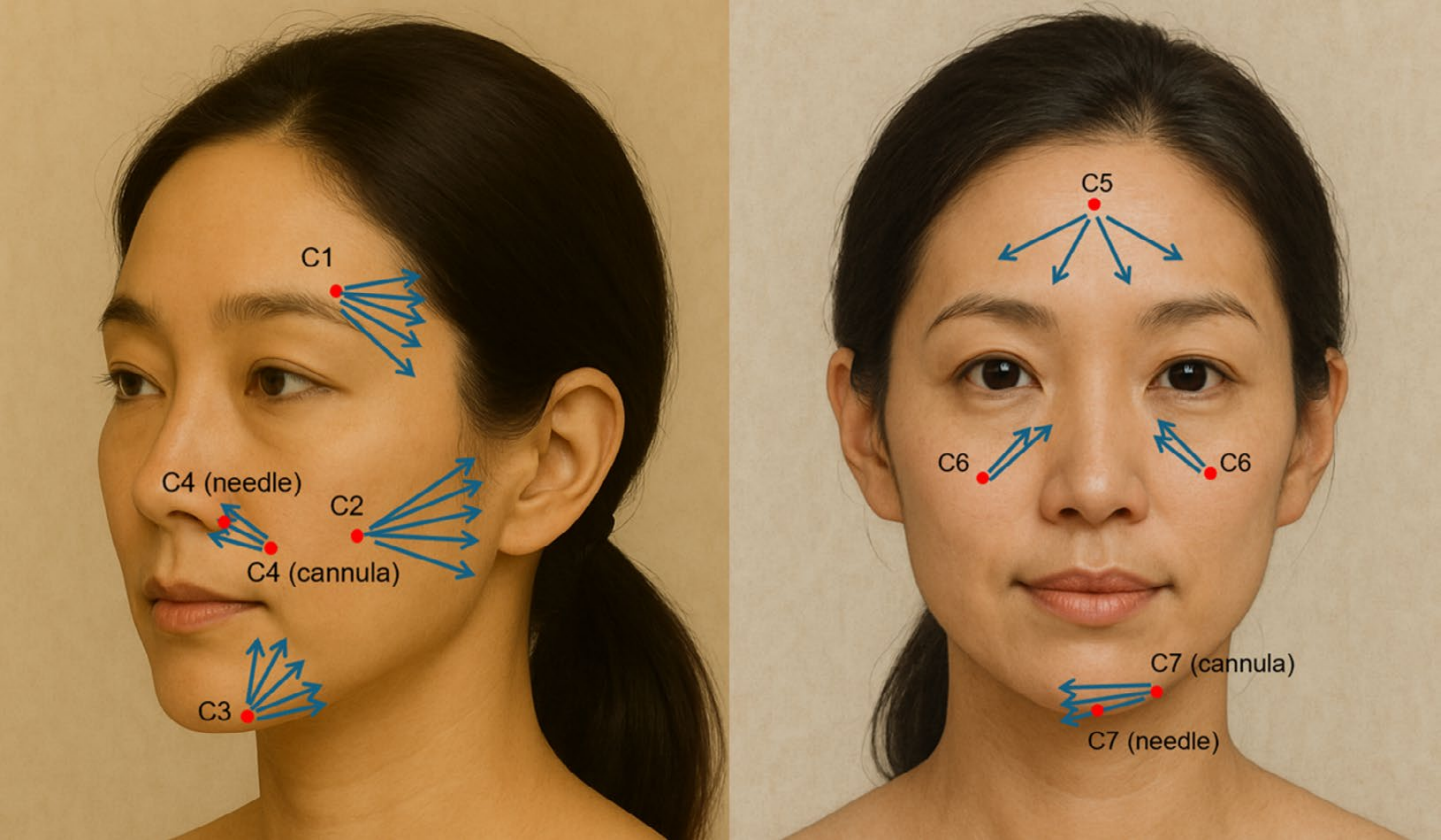
This schematic illustrates the contouring phase using polycaprolactone filler, with red dots marking entry points (C1–C7) and blue arrows indicating injection patterns.

To address this limitation, we developed the CL Code protocol, a dual‐phase injectable approach that aligns the temporal behavior of PCL with anatomically targeted treatment objectives [8]. This method integrates:

C‐phase (Contouring):

Utilization of the transient volumetric effect of the CMC carrier to correct volume deficits (Figure 1).

**FIGURE 2:**
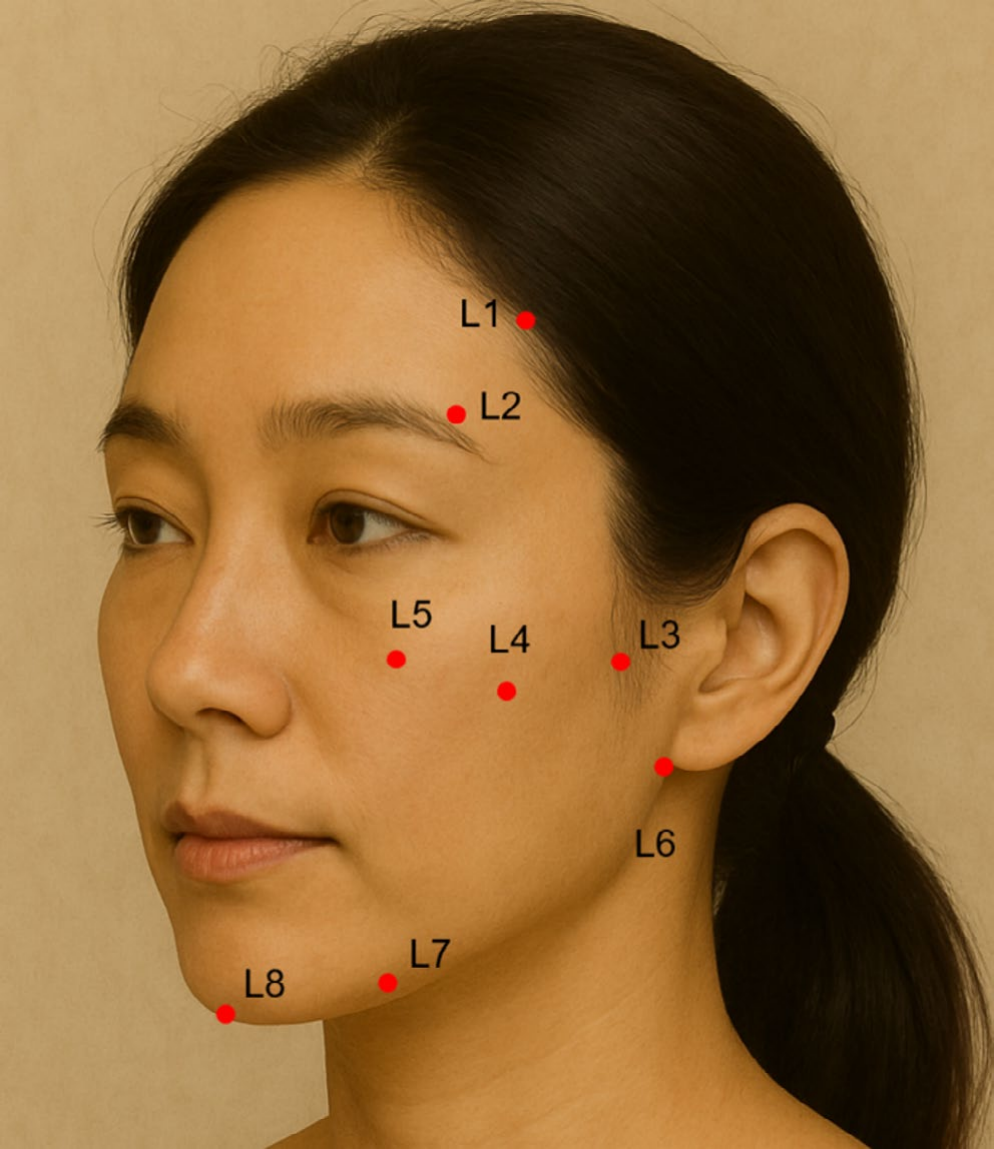
This schematic illustrates the ligamentous strengthening phase using polycaprolactone filler, with red dots marking entry points (L1–L8) and blue arrows indicating injection patterns.

L‐phase (Ligamentous Support):

Targeting of the sustained collagen‐stimulating activity of PCL microspheres toward the deep origin of the retaining ligaments (Figure 2).

Product formulation is selected according to tissue‐specific biointegration kinetics, with shorter‐duration PCL formulations applied during the contouring phase and longer‐duration formulations used for ligamentous anchoring. This temporal and anatomical stratification is intended to address both immediate volume loss and progressive structural support deficits associated with facial aging.

This article describes the anatomical rationale, technical execution, and 6‐month clinical outcomes of the CL Code protocol, including a representative case.

## 2 Facial Ligaments and Aging

The human face maintains its architectural integrity through an organized system of fibrous connections that link superficial soft tissues to underlying skeletal structures [2, 9, 10, 11]. Histological studies have characterized these connections as dense collagen bundles originating from periosteal surfaces and extending across multiple tissue planes. Although their microstructural organization differs from that of articular ligaments, these fibrous systems serve analogous mechanical functions by providing three‐dimensional stabilization of facial soft tissues [12, 13, 14].

Advances in medical imaging have enabled detailed mapping of these fibrous attachments, revealing consistent anatomical patterns across individuals. Clinically, these zones correspond to regions of restricted tissue mobility, where firm osseous attachments define natural boundaries between facial esthetic subunits. This structural arrangement provides a rational basis for targeted anatomical interventions. The biomechanical response to subfascial biostimulatory agents follows a predictable sequence, beginning with immediate volume expansion within the fibrous network, followed by progressive extracellular matrix remodeling [13]. This temporal pattern correlates with clinically observed changes in which early tissue displacement caused by the carrier gel precedes gradual increases in tissue tensile strength, ultimately resulting in sustained elevation and stabilization of overlying soft tissues. Such mechanical behavior is consistent with established principles of soft tissue engineering, whereby selective reinforcement of load‐bearing structures enhances overall system stability.

Clinical–anatomical studies have identified specific ligamentous zones of therapeutic relevance across different facial regions. In the upper face, the temporal region contains two primary stabilization points: the superior temporal septum, which consistently attaches along the temporal crest, and the temporal ligament adhesion, which serves as a key anchoring structure for the superficial temporal fascia [15]. In the midface, structural support is derived from three interconnected components: the zygomatic retaining ligament along the inferior border of the zygomatic arch, the zygomatic ligament proper contributing to anterior projection support, and the zygomaticocutaneous ligament influencing nasolabial contour formation [16]. The lower face relies on a coordinated ligamentous framework to maintain contour and resist gravitational descent. The platysmal–auricular connection provides lateral mandibular support, while the mandibular ligament complex acts as the principal stabilizer against jowl formation [17]. The mentalis anchoring complex, consisting of the mandibulocutaneous ligament and associated fascial attachments, stabilizes chin position by tethering soft tissue to the mandible. This integrated anatomical framework underpins systematic approaches to ligament‐targeted facial reinforcement. STS and TLA are considered false retaining ligaments; therefore, our ligament‐targeted concept is directed toward the deep origin of true retaining ligaments for structural reinforcement.

Treatment Protocol The CL Code protocol employs a dual‐phase strategy combining volumetric contouring (C Code) and ligamentous reinforcement (L Code). Detailed injection parameters and anatomical targets are summarized in Tables 1 and 2. All L Code injections are performed at the supraperiosteal or subsuperficial musculoaponeurotic plane using 27‐gauge needles.

**TABLE 1:** C‐Code Injection Parameters for Facial Contouring.

Code	Target area	Entry point	Depth/Layer	Volume (cc)
C1	Temporal hollow	1 cm above lateral eyebrow	STF‐DTF interface	0.3–0.7
C2	Lateral cheek hollow	Anterior to depression	SubSMAS	0.3–0.7 × 2
C3	Prejowl sulcus	Chin‐mandible junction	Supraperiosteal (borderline)	0.3–0.5 × 2
			SubSMAS (labiomandibular fold)	
C4a	Mild nasolabial fold	5 mm lateral to lower 1/3 crease	SubSMAS	0.3–0.5 × 2
C4b	Deep canine fossa	Central triangular depression	Ristow's space	0.3–0.5
C5	Forehead depression	Midforehead above depression	Submuscular	0.3–0.7
C6	Midcheek groove	Lateral to depression	SubSMAS	0.3–0.5 × 2
C7a	Mild chin depression	Central chin	SubSMAS	0.3–0.5
C7b	Severe chin depression	Chin‐mandible boundary	SubSMAS	0.3–0.7

**TABLE 2:** C‐Code Injection Parameters for Ligamentous Reinforcement.

Code	Target ligament	Entry point	Depth	Volume (cc)
L1	Superior temporal septum	Hairline at forehead‐temporal junction	Supraperiosteal	0.1
L2	Temporal ligament adhesion	Above lateral eyebrow	Supraperiosteal	0.1
L3	Posterior zygomatic ligament	Inferior to posterior zygomatic arch	SubSMAS	0.1
L4	Anterior zygomatic ligament	Inferior to anterior zygomatic arch	SubSMAS	0.1
L5	Zygomatic cutaneous ligament	Lateral midcheek groove	Supraperiosteal	0.1
L6	Platysma‐auricular ligament	Earlobe‐cheek junction	SubSMAS	0.1
L7	Mandibular ligament	Anterior mandibular border	Supraperiosteal	0.1
L8	Mental ligamentous complex	Central chin border	Supraperiosteal	0.1

C Code Technique:

For natural contour restoration, Ellansé‐S (Sinclair Pharma, London, United Kingdom) is administered at seven predefined anatomical zones, as outlined in Table 1.

L Code Technique:

Ligamentous anchoring is performed using Ellansé‐M (Sinclair Pharma, London, United Kingdom), targeting eight osteocutaneous support points described in Table 2.

## 3 Case Study

**FIGURE 3:**
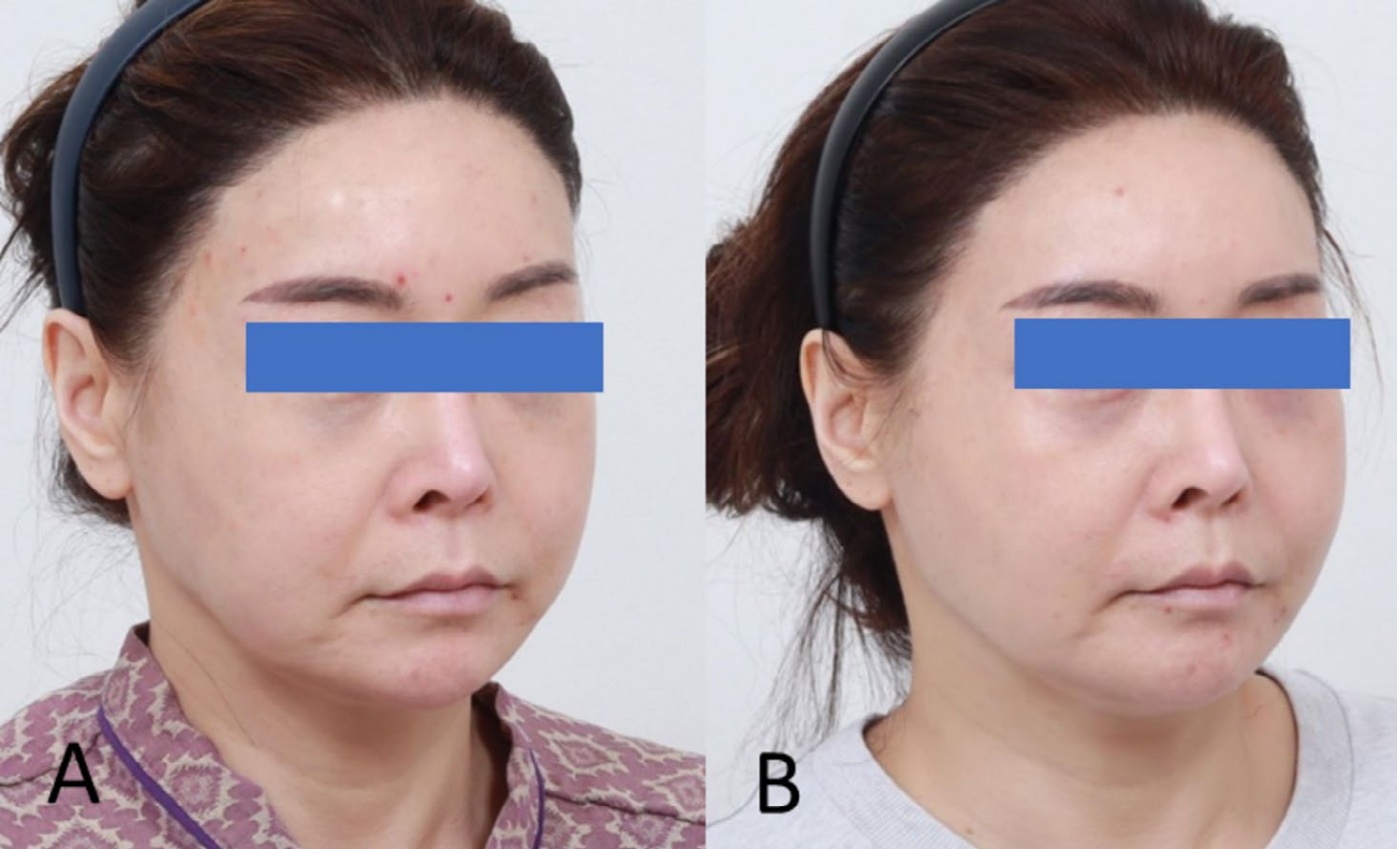
CL code treatment outcomes in 49‐year‐old female. (A) Baseline presentation demonstrating pan‐facial volume depletion with temporal hollowing, midcheek deflation, and mandibular border effacement. (B) Six‐month post‐treatment results showing improved jawline definition, reduced jowling, and softened nasolabial folds following administration of 2.9 mL total volume polycaprolactone filler.

**FIGURE 4:**
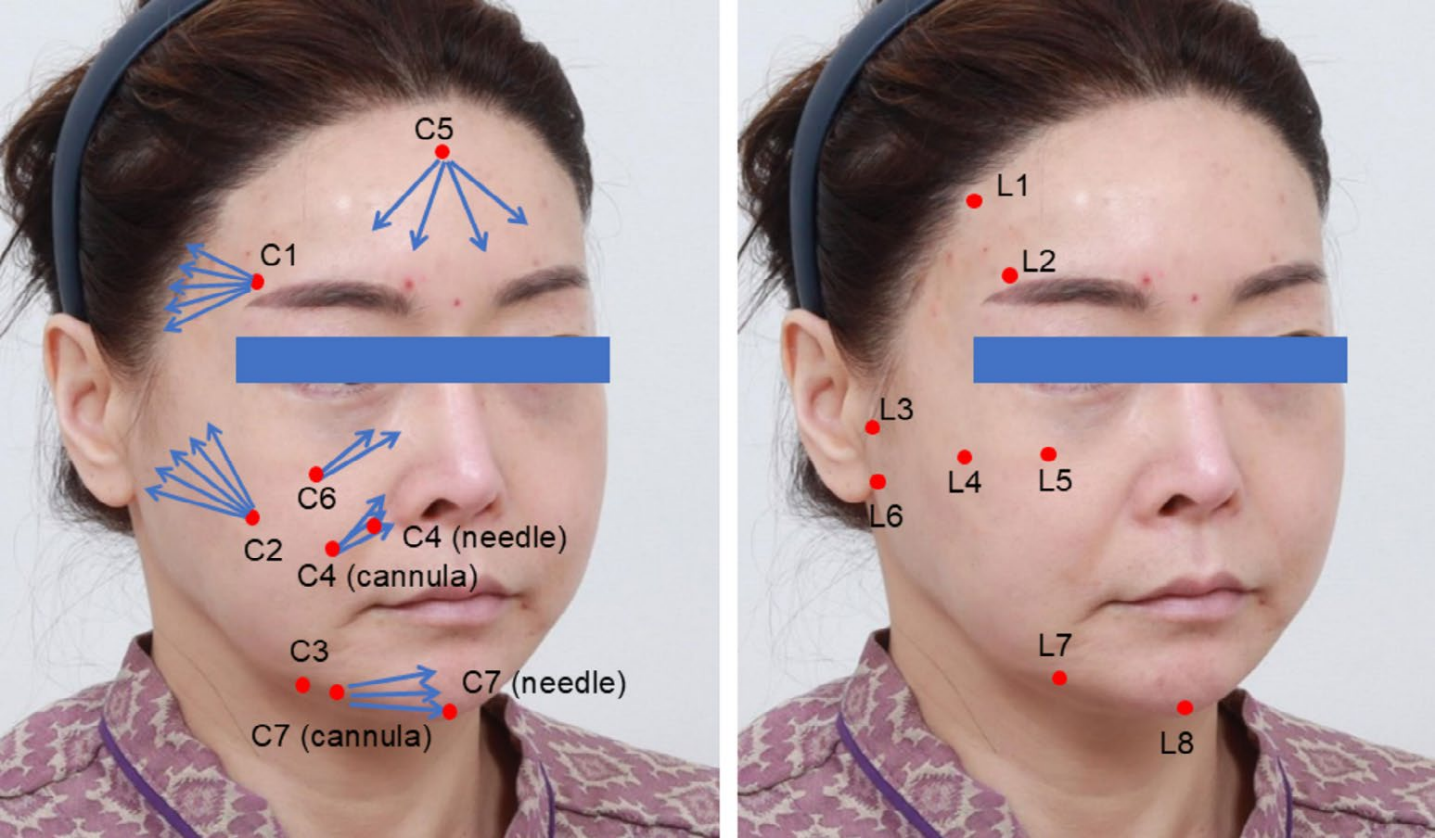
Patient photograph marking C‐Code and L‐Code entry points (red dots) and injection patterns (blue arrows).

A 49‐year‐old woman presented with pan‐facial volume depletion, including temporal hollowing, midcheek deflation, and loss of mandibular definition (Figure 3A). Treatment was performed according to the CL Code protocol (Figure 4; Tables 1 and 2), with 0.3 mL aliquots of Ellansé‐S (Sinclair Pharma, London, United Kingdom) administered across seven contouring zones (C1–C7), complemented by 0.1 mL deposits of Ellansé‐M placed at eight ligamentous support points (L1–L8). At the 6‐month follow‐up, standardized photographic evaluation demonstrated visible improvement in jawline definition, reduction of jowling, and softening of nasolabial folds (Figure 3B). Global Aesthetic Improvement Scale (GAIS) assessment by two independent dermatologists rated the outcome as “Very Much Improved.” Patient‐reported FACE‐Q scores increased from 58 to 86 (out of 100), indicating high satisfaction.

## 4 Safety

No adverse events were reported during the 6‐month follow‐up period. Immediate postinjection edema and mild ecchymosis resolved within 3–5 days. The procedure was well tolerated with topical anesthesia.

## 5 Discussion

This case evaluates 6‐month outcomes following treatment with the CL Code protocol in a 49‐year‐old female patient. The intervention employed a total volume of 2.9 mL for full‐face correction, representing a conservative approach compared with conventional filler techniques, which often require larger volumes for comprehensive facial rejuvenation [18]. Clinical assessment demonstrated improved facial contour, potentially attributable to targeted reinforcement of ligamentous attachment points. This interpretation is supported by anatomical studies highlighting the biomechanical significance of retaining ligaments in maintaining facial structural support [19].

Clinical observations suggest that selective reinforcement of key ligamentous attachments may contribute to improved facial contour; however, the single‐case design precludes broader generalization. The 6‐month follow‐up period corresponds to the early phase of PCL‐induced collagen stimulation, while longer‐term studies are necessary to evaluate durability beyond this interval [4, 7]. Future investigations should include controlled comparisons with conventional filler techniques, standardized volumetric analyses, and evaluation of optimal injection volumes tailored to specific facial regions. This report contributes to the evolving understanding of anatomy‐guided strategies for facial rejuvenation using collagen‐stimulating agents. Specifically, the study cannot distinguish the relative contributions of immediate volumization (C‐phase) versus ligamentous reinforcement (L‐phase). In addition, imaging confirmation of anatomical targeting was not performed. The 6‐month follow‐up represents an early stage of PCL‐induced neocollagenesis (typically 12–24 months), and longer‐term follow‐up is needed to assess durability.

## 6 Conclusion

The CL Code protocol integrates targeted volumization (C Code) and ligamentous reinforcement (L Code) using PCL‐based fillers to address the multifactorial mechanisms of facial aging. This report suggests that the approach can achieve improved facial contour and high patient satisfaction at 6 months using conservative injection volumes (2.9 mL). Controlled comparative studies are warranted to further validate its efficacy relative to conventional filler techniques.

## Author Contributions

All authors have reviewed and approved the article for submission. Conceptualization: Gi‐Woong Hong, Isaac Kai Jie Wong, Jovian Wan, Soo Yeon Park, Suyeon Lee, Kyu‐Ho Yi. Writing – original draft preparation: Jovian Wan, Gi‐Woong Hong, Isaac Kai Jie Wong, Soo Yeon Park, Suyeon Lee, Kyu‐Ho Yi. Writing – review and editing: Jovian Wan, Gi‐Woong Hong, Isaac Kai Jie Wong, Soo Yeon Park, Suyeon Lee, Kyu‐Ho Yi. Visualization: Gi‐Woong Hong, Isaac Kai Jie Wong, Jovian Wan, Soo Yeon Park, Suyeon Lee, Kyu‐Ho Yi. Supervision: Kyu‐Ho Yi.

## Funding

The authors have nothing to report.

## Ethics Statement

This study was conducted in accordance with the principles of the Declaration of Helsinki. Ethical approval was not required for this case‐based technical report. Written informed consent was obtained.

## Consent

Written informed consent was obtained from the patient prior to treatment. Written informed consent for publication of clinical images was obtained from the patient.

## Conflicts of Interest

The authors declare no conflicts of interest.

## Data Availability

The data that support the findings of this study are available from the corresponding author upon reasonable request.
